# The Effect of Hunger and Satiety on Mood-Related Food Craving

**DOI:** 10.3389/fpsyg.2020.568908

**Published:** 2020-11-02

**Authors:** Janina Reents, Ann-Kathrin Seidel, Christian Dirk Wiesner, Anya Pedersen

**Affiliations:** Department of Clinical Psychology and Psychotherapy, Institute of Psychology, Christian-Albrechts-University zu Kiel, Kiel, Germany

**Keywords:** mood induction, normal weight, eating behavior, cue-reactivity paradigm, cue-reactivity, emotional eating

## Abstract

In addition to hunger, which results from food deprivation, fasting, or restrained eating, food craving is thought to be one of the major factors influencing eating behavior. Although food craving occurs in healthy humans with normal eating behavior, research suggests pronounced food craving to be a risk factor for binging food, which in turn might cause weight gain and obesity. In addition, negative mood seems to induce binge eating, although the underlying processes are not clearly understood. To examine these effects on momentary food craving more closely, we implemented a food cue-reactivity paradigm in 33 normal-weight females (mean age = 25.1 years, mean BMI = 21.6). Mood (negative vs. neutral) and state of food deprivation (hunger vs. satiety) were varied systematically. Self-rated craving was significantly higher in hungry compared to sated states. Moreover, in neutral mood, high-caloric foods modulated craving, i.e., hungry and sated participants preferred savory food and sweet food, respectively. In negative mood, this differential effect of savory and sweet items was absent. In summary, the state of hunger has a huge impact on food craving, which is further modulated by emotional state. Future research should consider hunger to be of high relevance for effects investigated in food-related paradigms. Our findings in healthy women of normal weight support etiological models of disturbed eating behavior and are in line with common interventions targeting eating schedules and mood regulation.

## Introduction

Craving food, i.e., perceiving an intense desire for specific food, is a common experience in healthy persons; hence, it is not limited to an exaggerated or pathological urge to eat but is also an important part of normal eating behavior (Weingarten and Elston, 1990; Hill, 2007). As a motivational state, food craving is understood as a conditioned response to sensory, situational, or interoceptive cues (Wardle, 1990; Finlayson et al., 2007). Since food cravings mostly refer to calorie-dense, highly palatable food (Pelchat and Schaefer, 2000), research suggests pronounced food craving to be a risk factor for binging food, which in turn might cause weight gain and obesity (Sobik et al., 2005; Ng and Davis, 2013). More specifically, the trait version but not the state version of the Food Cravings Questionnaire (FCQs German version; Meule et al., 2012) discriminated between successful and unsuccessful dieters (Meule et al., 2012). However, the state version was affected by other underlying states like mood and hunger.

The biological state of hunger is obviously one of the most prominent factors influencing eating behavior (Finlayson et al., 2007; Dalton and Finlayson, 2014; Andermann and Lowell, 2017). For example, in normal-weight subjects as well as in individuals with obesity, hunger generates an attentional bias towards food cues (Loeber et al., 2012, 2013). This attentional bias consists of a decreased response inhibition towards food-associated stimuli when subjects are hungry. However, it remains unclear how self-perceived hunger influences the processing of food stimuli, when attention is focused. Moreover, it is unknown how hunger affects explicit food craving at the sight of highly palatable foods. Although the state food craving assessed *via* questionnaire is found to be positively correlated with hours elapsed since the latest meal (Meule et al., 2012), it remains unclear how this accounts for the explicit reactivity at the sight of food.

Another major factor influencing state food craving and eating behavior is the emotional state, which is suggested to have both an increasing and decreasing effect on food intake (Macht, 2008). Supporting evidence for such a mood-modulated effect on food intake was revealed in a recent meta-analysis by Cardi et al. (2015), who found negative mood states such as dysphoria or anxiety to be linked to higher food intake in restrained eaters and subjects with binge eating disorder. In addition, positive emotional states were associated with a greater caloric intake in healthy controls, although the results were less clear-cut (Cardi et al., 2015). In this context, emotions themselves seem to become conditioned stimuli and elicit appetitive responses (Wardle, 1990; Bongers et al., 2015). Furthermore, this mood-dependent impact on food intake seems to be modulated by underlying habitual aspects of mood regulation. For instance, Jansen et al. (2008) demonstrated two distinct subtypes of persons with overweight, i.e., the “high negative affect” subtype and the “low negative affect” subtype, which are differentially prone to the induction of negative mood, resulting in higher food consumption in the high negative affect subtype only. Furthermore, a trait-like style of food craving in response to negative stimuli, which is labeled “high emotional eating” vs. “low emotional eating,” has been shown to be a valid construct in lean female subjects (Blechert et al., 2014a). Although hunger is not understood as a precondition for food craving, it is still an important predictor of its intensity (Nederkoorn et al., 2000; Hill, 2007). Accordingly, Blechert et al. (2014a) instructed their participants to abstain from any food or caloric drinks 3 h prior to the laboratory assessments of food craving to control for hunger at the time of testing.

Systematic studies on the influence of hunger on food cues are sparse. They mostly concentrate on the implicit effects of hunger on attention to food cues and findings are inconsistent. For instance, Castellanos et al. (2009) manipulated hunger in an eye-tracking paradigm and revealed an attentional bias for food cues in a fasted state in lean individuals and participants with obesity. However, this attentional bias vanished after food intake in the normal-weight controls, whereas participants with obesity maintained a gaze direction bias for food over nonfood stimuli even in a state of satiety. However, in the same study, there was no significant effect of hunger on reaction times towards food stimuli and no food-associated reaction time bias. Loeber et al. (2013) revealed hunger to affect behavioral response inhibition in a visual probe task, indicated by enhanced attention allocation in response to food-associated cues in normal-weight controls as well as in participants with obesity. In addition, decreased inhibitory control in a Go/No-Go task was revealed when subjects performed in a hungry state (Loeber et al., 2013).

To the best of our knowledge, an experimental variation of hunger and satiety in the case of cue-reactivity to food stimuli has been rarely realized (Führer et al., 2008; Castellanos et al., 2009; Goldstone et al., 2009), although even adhering to a monotonous diet leads to differential effects in behavioral measurements of state food craving (Polivy et al., 2010; Ledoux et al., 2013). Instead of systematically varying the state of hunger, it is a common procedure to implement a more or less homogeneous fasting duration. For instance, numerous recent studies have asked participants to adhere to a period of food deprivation ranging from 1 to 18 h before the assessment (Sobik et al., 2005; Jansen et al., 2008; Martin et al., 2008; Mahler and de Wit, 2010; Doolan et al., 2014; Brockmeyer et al., 2015; Mühlberg et al., 2016). This procedure involves a large range of fasting hours not only between studies but also often within samples. Moreover, many different approaches are implemented to assess the state of hunger, e.g., participants estimate the elapsed time since their latest meal or rate their perceived hunger in different self-ratings (Meule et al., 2014; Hume et al., 2015; Kemps et al., 2016). Occasionally, these measures are accomplished with an assessment of blood glucose levels (Loeber et al., 2013). Interestingly, blood glucose levels and self-reported hunger show inhomogeneous associations with behavioral measures such as attention allocation to food-associated cues and inhibitory control (Loeber et al., 2013). Hence, blood glucose levels and perceived hunger might reflect different processes controlling food intake, whereas the authors have emphasized that self-reported hunger is particularly relevant for the perception of food-related stimuli (Loeber et al., 2013). Corresponding effects, i.e., a higher vigilance for sweet food, have been revealed even when women with overweight and obesity report feeling only slightly hungry (Gearhardt et al., 2012).

The aim of the present study was to evaluate the effect of hunger and satiety on mood-related state food craving with cue-reactivity, taking particular account of aversive mood states such as dysphoria. As self-reported hunger is linked to an increased salience of food cues, we expected fasting to increase food cue-reactivity and state food craving (Mogg et al., 1998; Stockburger et al., 2009; Meule et al., 2014). Therefore, this effect should be strongest for negative mood, as dysphoria has been linked to higher salience of food stimuli as well as higher food intake. Moreover, we expected food stimuli to be generally preferred over nonfood stimuli.

## Materials and Methods

### Participants

We calculated an *a priori* total sample size with an estimated effect of *η*^2^ = 0.1 based on the findings of Ledoux et al. (2013) and the meta-analysis by Cardi et al. (2015). To detect the hypothesized within–between interaction at *α* = 0.05 with a power of 0.95, we found the minimum required sample size to be *n* = 22 using G*Power (Faul et al., 2007). We also conducted a *post hoc* power analysis of the three-way interaction using the generic *F* test option in G*Power (Faul et al., 2007), which indicated a quite high power of at least 0.878.

A total of 36 normal-weight females aged between 19 and 49 years participated in the study. They were recruited by posters at the University of Kiel, asking for healthy, female volunteers, who habitually ingest breakfast. In a standardized telephone interview, respondents were screened for the following exclusion criteria: body mass index (BMI) below 19 or over 25, menopause, severe mental illness (including eating disorders), taking psychoactive medication, pregnancy or breastfeeding, vegan or vegetarian diet, or suffering from food intolerances. However, due to high hunger levels in satiety condition, we had to exclude two participants and one subject did not show up for the second assessment. Therefore, our final sample consisted of 33 subjects. The mean age of the included participants was 25.1 years (*SD* = 6.0), and their mean BMI was 21.6 (*SD* = 1.5, range: 19–24.6). The participants had a mean score of depression symptom severity of 3.61 (*SD* = 3.29) in the Beck Depression Inventory (BDI-II German version; Beck et al., 2001). Their mean of psychopathological eating behaviors in the Eating Disorder Examination Questionnaire (EDE-Q; Hilbert and Tuschen-Caffier, 2006) was 0.59 (*SD* = 0.44). None of them reported a pathological amount of binge eating episodes (referring to DSM-5 criteria, an amount of at least two times per week within the last month, i.e., 8 days within the past 28 days, is regarded as critical; American Psychiatric Association, 2013) or inappropriate weight compensatory behavior (no one reported use of laxatives or vomiting for weight control). Subjects had a mean of 80.54 (*SD* = 20.74) in the Food Cravings Questionnaire Trait (FCQ-T; Meule et al., 2012). There is evidence that food craving is enhanced during the luteal phase of the menstrual cycle in women who are not taking hormonal contraceptives (Gorczyca et al., 2016). Nine of our 33 participants were not taking hormonal contraceptives and had a regular cycle. As we identified only three of these subjects to be in the luteal phase at the first assessment and one at the second assessment, the influence seems to be less relevant in this sample. Further information is provided in Table 1. The study adhered to the declaration of Helsinki. All participants provided written informed consent and received 20€ for their participation in the study.

**Table 1:** Sample characteristics separated by between-group factor mood.

	Negative mood group (*n* = 15)	Neutral mood group (*n* = 18)	Test statistics
Age (years)	*M* = 25.4, *SD* = 4.6	*M* = 24.8, *SD* = 7.1	*t*(31) = 0.27, *p* = 0.793
BMI	*M* = 21.2, *SD* = 1.5	*M* = 22.0, *SD* = 1.3	*t*(31) = −1.51, *p* = 0.142
BDI-II	*M* = 3.2, *SD* = 3.1	*M* = 3.9, *SD* = 3.5	*t*(31) = −0.64, *p* = 0.527
EDE-Q	*M* = 0.48, *SD* = 0.33	*M* = 0.68, *SD* = 0.51	*t*(31) = −1.29, *p* = 0.207
Number of subjects reporting binge days in the past 28 days	*n* = 0	*n* = 3^a^	-
Number of subjects reporting use of laxatives or vomiting	*n* = 0	*n* = 0	-
FCQ-T	*M* = 84.8, *SD* = 24.19	*M* = 77.0, *SD* = 17.3	*t*(31) = 1.08, *p* = 0.289

M, mean; SD, standard deviation; BMI, body mass index; BDI-II, Beck Depression Inventory-II; EDE-Q, Eating Disorder Examination Questionnaire; FCQ-T, Food Craving Questionnaire Trait.

a One subject reported one binge eating day, one reported 2 days of binge eating, and one reported 3 days of binge eating.

### Self-Report Measurements of Eating Behavior

The German version of the Food Cravings Questionnaire State (FCQ-S; Meule et al., 2012) measures momentary desire for specific foods. The questionnaire consists of 15 items that are assessed on a five-point scale ranging from 1 = “strongly disagree” to 5 = “strongly agree.” The FCQ-S comprises five subscales: desire, positive reinforcement, negative reinforcement, lack of control, and hunger. The FCQ-S has a high internal consistency both for the total value (Cronbach’s *α* = 0.92) and for the subscales (0.87≤ Cronbach’s *α* ≤ 0.89).

The German version of the Food Cravings Questionnaire Trait (FCQ-T; Meule et al., 2012) measures food craving as a trait-like desire for food. The questionnaire consists of 39 items, which are scored on a six-point scale ranging from 1 = “never” to 6 = “always.” The FCQ-T comprises nine subscales: intentions, positive reinforcement, negative reinforcement, lack of control, thoughts, hunger, emotions, cues, and guilt. The internal consistency of the FCQ-T can be rated as good for the overall sum (Cronbach’s *α* = 0.96) and the subscales (0.72 ≤ Cronbach’s *α* ≤ 0.93).

The German version of the Eating Disorder Examination Questionnaire (EDE-Q; Hilbert and Tuschen-Caffier, 2006) measures pathological eating behaviors and related concerns. It comprises four subscales: restraint, eating concern, weight concern, and shape concern. The EDE-Q has good convergent validity and the questionnaire is able to differentiate between persons with and without eating disorders. Accordingly, a mean sum of 1.44 with *SD* = 1.22 is regarded as normal for subjects without eating disorders (Hilbert and Tuschen-Caffier, 2006). Both the four subscales and the total value have high internal consistencies (subscales: 0.85 ≤ Cronbach’s *α* ≤ 0.93; total value: Cronbach’s *α* = 0.97).

### Design and Procedures

*Via* experimental manipulation of neutral vs. negative mood, we set up two groups of participants. The participants were randomly allocated to these two groups and did not differ in BMI, trait food craving, eating behavior, and eating psychopathology or depression (see Table 1). All participants completed two separate sessions, and in both sessions, the primarily allocated mood was induced again. Therefore, the mood condition was the same in both sessions for each subject, but the state of food deprivation was varied: the first session was conducted in a hungry state, and the second one was conducted in a sated state.

To induce hunger, we instructed all participants to partake in their habitual breakfast between 8:30 and 9 AM on the day of the first session and to fast for 6 h until the scheduled testing at 3 PM. During this fasting period, participants were also asked to refrain from consuming beverages, except for a maximum of 2 L of water. When arriving at our laboratory, the participants first reported their latest meal and drink and then rated their perceived hunger on a visual analog scale (ranging from “not at all hungry” to “very hungry”).

In the second session, participants adhered to a sated state. They were instructed to consume a normal portion of a usual meal without a dessert between 12:30 and 1 PM on testing day and to refrain from eating until 1 h before testing at 2 PM (for example, subjects reported “fish fingers and salad” and “ratatouille with pasta”). If instructions were not followed, the assessments would have been postponed, but this did not occur. To prevent memory, order, or carry-over effects, we introduced an exceptionally long pause of 3–6 months between sessions (*M* = 20.1 weeks, *SD* = 4.5 weeks). Both sessions followed the same procedure. Throughout both sessions, a researcher was present to ensure task compliance and to answer the participant’s questions.

#### Mood Induction

We implemented the mood induction task by Robinson et al. (2012) to induce either negative or neutral mood as a between-subjects factor. In the neutral mood condition, participants read 60 neutral Velten sentences (Velten, 1967) while listening to The Planets, Op. 32: VII Neptune, the Mystic by Holst (n.d.). In the negative mood condition, participants were asked to select the saddest piece of music between Adagio for Strings, Op. 11 by Barber (n.d.) and Adagio in G Minor by Albinoni (n.d.) and to read the set of 60 negative Velten sentences while listening to the chosen piece. The music was presented in a loop *via* full-ear headphones, while the corresponding sentences were presented consecutively on screen. As each sentence was presented for 12 s, this procedure took 12 min. Mood induction was implicated before the computerized cue-reactivity paradigm and maintained by presenting the same music in a loop. In a dysphoria rating, the participants evaluated their current mood on a visual analog scale ranging from “not at all sad” to “very sad” directly after the mood induction procedure (pre) and again after the cue-reactivity paradigm (post).

#### Food Cue-Reactivity

**Figure 1:**
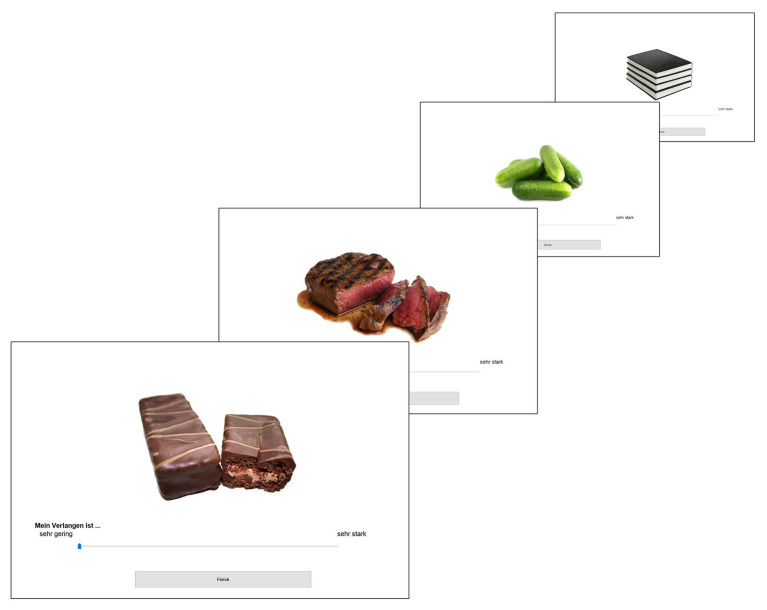
Examples for each category of stimuli (sweet, savory, raw, and nonfood) in our cue-reactivity paradigm with visual analog rating scale; Pictures from the food-pics image database (Blechert et al., 2014b).

The cue-reactivity paradigm was developed in our laboratory and is comparable to other paradigms that measure food cue-reactivity *via* pictorial content (Blechert et al., 2014a), i.e., the participants were asked to view pictures and rate their cravings. We selected 200 pictures from the image database of experimental research on eating and appetite (Blechert et al., 2014b) and built four subsets of 50 pictures each. The sweet food pictures depicted food items with high fat/high sugar content (e.g., chocolate cake). Likewise, pictures of the savory food category depicted food items with a high amount of fat and carbohydrates and/or salt (e.g., hamburger and French fries). Pictures in the raw food category included food items with a modest calorie content (e.g., raw fruits, vegetables, and crispbread). The 50 nonfood pictures depicted inedible objects (e.g., screws, light bulb). All pictures were presented on a computer screen that showed the stimulus centered on a white background using Inquisit 4 (2015). The participants were asked to look at the presented picture for at least 1 s and then rate their currently perceived craving to consume the shown food item or – as in the case of nonfood stimuli – to engage with the item. The rating was given by moving the mouse pointer on a visual analog rating scale (VAS) displayed beneath the stimulus that was labeled as “my craving is” and ranged from “very low” to “very high.” The stimulus stayed presented on the screen until the participant hit a “finish” button beneath the VAS. Examples are shown in Figure 1.

### Statistical Analysis

All analyses were performed using IBM Corp. Released 2013 (n.d.). We conducted *t* tests to control for group differences in demographical and questionnaire data (see Table 1). To determine the effectiveness of our instructions, we checked hunger (self-reported state of hunger) before the cue-reactivity paradigm in both sessions (first session in a hungry state vs. second session in a sated state). For the same reason, we assessed mood before and after the paradigm (dysphoria rating pre vs. post cue-reactivity paradigm) in both sessions.

We conducted mixed ANOVAs for our 2 × 2 × 4 design with state of hunger as a within-subjects factor (hungry vs. sated state), mood as a between-subjects factor (negative vs. neutral), and stimulus category as a within-subjects factor (raw food, sweet food, savory food, and nonfood). Significance was assumed at *p* < 0.05. When required, degrees of freedom were adjusted with the Greenhouse-Geisser correction for nonsphericity.

To assess food craving as a dependent variable, we calculated means of the craving ratings assessed in the cue-reactivity paradigm for the four categories and each participant. To further validate that our paradigm assessed state food craving, we asked the participants to fill out the FCQ-S before and after the cue-reactivity paradigm.

For the significant effects in our main analysis, *post hoc t* tests were conducted.

## Results

### Dysphoria Rating

As a manipulation check of the mood induction, we computed an ANOVA on the dysphoria ratings with the within factors state of hunger (hungry vs. sated) and time of measurement (pre vs. post cue-reactivity paradigm) and the between factor mood induction (negative vs. neutral). As expected, the analysis revealed a main effect of mood induction, *F*(1, 31) = 9.09, *p* = 0.005, *η*^2^ = 0.23, and a main effect of state of hunger, *F*(1, 31) = 8.04, *p* = 0.008, *η*^2^ = 0.21, but no main effect of time of measurement and no significant interaction effects. *Post hoc t* tests confirmed a higher level of dysphoria in the negative mood condition as compared to the neutral mood condition in hungry participants (negative: *M* = 4.74, *SD* = 2.54; neutral: *M* = 2.76, *SD* = 2.54) with *t*(31) = 2.22, *p* = 0.034, *d* = 0.78, as well as in sated participants (negative: *M* = 3.95, *SD* = 1.92; neutral: *M* = 1.89, *SD* = 2.31) with *t*(31) = 2.75, *p* = 0.010, *d* = 0.96.

The effect of mood induction was stable as the ANOVA showed no significant main effect and additional paired *t* tests revealed no significant difference between the pretest and the posttest on the dysphoria ratings within the groups neither in a sated state, negative mood condition: *t*(14) = 1.66, *p* = 0.120; neutral mood condition: *t*(17) = 0.92, *p* = 0.371, nor in a hungry state, negative mood condition: *t*(14) = 0.03, *p* = 0.976; neutral mood condition: *t*(17) = −0.30, *p* = 0.769.

### Hunger Rating

As a manipulation check of the hunger induction, we computed an ANOVA with the within factor state of hunger (hungry vs. sated) and the between factor mood (negative vs. neutral). As expected, the analysis revealed a significant main effect of state of hunger, *F*(1, 31) = 628.53, *p* < 0.001, *η*^2^ = 0.95, indicating higher levels of self-reported hunger in a hungry state as compared to a sated state, but no significant main effect of mood and no interaction between state of hunger and mood. To make sure that both groups experienced comparable levels of hunger and that in each group the manipulation was successful, we computed additional *t* tests that confirmed the validity of our manipulation (see Table 2).

**Table 2:** Means, standard deviations, and results of *post hoc t* tests for hunger ratings.

Groups	Hungry State M (SD)	Sated State M (SD)	
Negative Mood	85.3 (10.6)	10.2 (13.9)	*t*(14) = 18.27, *p* < 0.001
Neutral Mood	81.9 (12.6)	8.6 (7.3)	*t*(17) = 17.56, *p* < 0.001
	*t*(31) = 0.82, *p* = 0.417	*t*(31) = 0.44, *p* = 0.666	

M, mean; SD, standard deviation.

#### Food Craving *via* Paradigm

**Figure 2:**
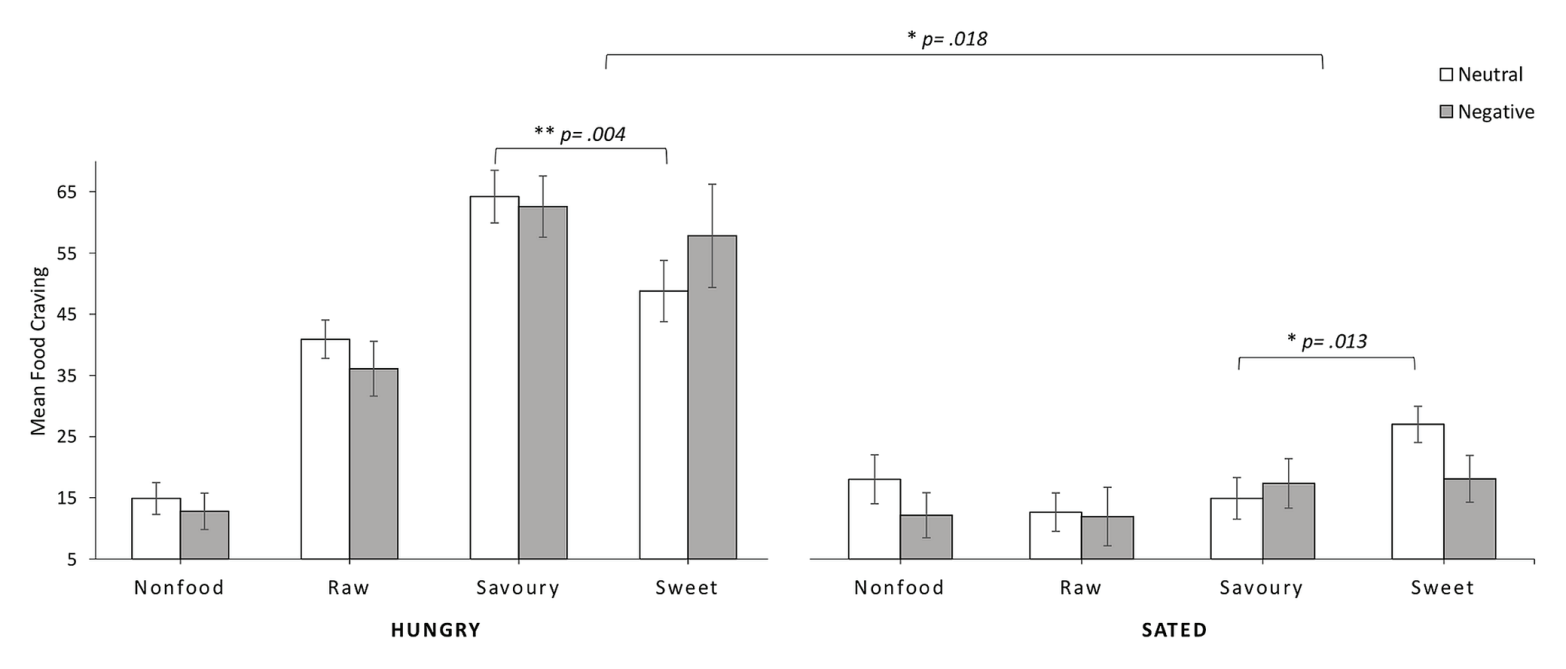
Results of mean state craving (±SEM) in the two states of hunger and satiety separated for neutral and negative mood group regarding the four stimulus categories.

We conducted a mixed ANOVA with the within-subject factors state of hunger (hungry vs. sated state) and stimulus category (raw food, sweet food, savory food, and nonfood) and the between-subject factor mood (negative vs. neutral). The analysis revealed a significant main effect of state of hunger, *F*(1, 31) = 264.54, *p* < 0.001, *η*^2^ = 0.90, i.e., participants in a hungry state, *M* = 49.04, *SD* = 14.34, reported more craving than participants in a sated state, *M* = 17.22, *SD* = 12.63 (see Figure 2). We also found a significant main effect of stimulus category, *F*(3, 31) = 24.30, *p* < 0.001, *η*^2^ = 0.44, indicating that participants showed differential reactions to the four categories of stimuli. We did not find a significant main effect of mood, *F*(1, 31) = 0.23, *p* = 0.637. Moreover, we found a significant interaction of food category × state of hunger, *F*(3, 31) = 52.53, *p* < 0.001, *η*^2^ = 0.63. *Post hoc* paired *t* tests revealed that participants favored food stimuli significantly over nonfood stimuli when hungry, *t*(32) = 10.52, *p* < 0.001, *d* = 2.71, but not when sated, *t*(32) = 0.52, *p* = 0.605. Our overall results are depicted in Figure 2.

A significant three-way interaction of state of hunger × mood × food category, *F*(3, 93) = 3.53, *p* = 0.018, *η*^2^ = 0.10, indicated that depending on the current state of hunger and current mood, participants responded differently to the four categories of stimuli presented (see Figure 2). *Post hoc t* tests revealed that hungry participants in neutral mood significantly preferred savory over sweet food, *t*(17) = 3.28, *p* = 0.004, *d* = 0.85, while the same participants in the sated state condition preferred sweet over savory food, *t*(17) = −2.77, *p* = 0.013, *d* = −0.74. For participants in the negative mood condition, the craving of savory and sweet nourishments did not differ significantly in a hungry state, *t*(14) = 0.67, *p* = 0.516, or a sated state, *t*(14) = −0.22, *p* = 0.829.

#### Food Craving *via* FCQ-S

Although our main dependent variable was the craving rating from the cue-reactivity paradigm, we sought to validate the rating using the sum of the FCQ-S. We conducted an ANOVA on the FCQ-S with the within factors state of hunger (hungry vs. sated) and time of measurement (pre vs. post cue-reactivity paradigm) and the between-group factor mood (negative vs. neutral). As expected, the analysis revealed a main effect of state of hunger, *F*(1, 31) = 278.11, *p* < 0.001, *η*^2^ = 0.90, indicating higher state food craving in the hunger condition, and a main effect of time of measurement, *F*(1, 31) = 23.24, *p* < 0.001, *η*^2^ = 0.428, indicating higher state food craving after the confrontation with food stimuli. The analysis revealed no main effect of mood induction and no interactions.

To further evaluate the differences from before and after the presentation of the food cues, we conducted additional *t* tests: in the hungry condition, paired *t* tests for the FCQ-S sum score revealed a significantly higher craving after the paradigm as compared to before (pre: *M* = 47.8, *SD* = 8.3; post: *M* = 52.0, *SD* = 10.0; *t*(32) = −3.45, *p* = 0.002, *d* = −0.45). Except for the subscale Desire, each subscale revealed a significant result with *p* ≤ 0.003. In the sated condition, paired *t* tests for the FCQ-S sum score revealed significantly higher craving after the paradigm as compared to before, pre: *M* = 21.9, *SD* = 6.2; post: *M* = 28.3, *SD* = 10.5; *t*(32) = −4.41, *p* < 0.001, *d* = −0.68. Except for the subscale Lack of Control, each subscale revealed a significant result with *p* ≤ 0.009. These results indicate that state food craving was elevated in both conditions when the subjects completed the questionnaire in a sated and a hungry state.

## Discussion

In the current study, we explored the interaction of mood and food saturation on subjective ratings of momentary craving in a food cue-reactivity paradigm involving different food and nonfood stimuli. For this purpose, we tested healthy females twice, first in a hungry state and then in a sated state, after the induction of either neutral or negative mood.

Our results show that, in general, hunger substantially elevated state food craving and contributed to a measurable preference for food over nonfood stimuli. Therefore, our study emphasizes the finding that food craving cannot be interpreted independently of participant’s self-perceived hunger (Castellanos et al., 2009; Goldstone et al., 2009; Stockburger et al., 2009; Hepworth et al., 2010; Donofry et al., 2019). In addition, discrepancies in state craving for sweet compared to savory food were established only in sated subjects but not in participants who had fasted before testing. In line with findings of increased salience of food cues and increased food cue-reactivity in food deprivation (Mogg et al., 1998; Stockburger et al., 2009), our results also emphasize the general impact of hunger on explicit measures of state food craving (Meule et al., 2014). On a behavioral basis, such differences in cue-reactivity might be reflected by healthy normal-weight eaters getting picky at the sight of food, only when they are sated. On a broader view and with regard to limitations due to the small sample size, our findings provide further evidence for the influence of sensory-specific satiety as recently evaluated for sweetness by Low et al. (2014). In their review, the authors describe the perceived blandness of usual meals compared to highly sweet and calorie-dense food. When the human brain recognizes this palatability, it even overrides satiety signals for consumption. This is commonly known as the “dessert stomach.”

To address not only food saturation but also the effects of emotional eating (e.g., Macht, 2008), cue-reactivity to food pictures in hunger and satiety was further investigated in different emotional states, as food-seeking and consuming might serve as behavior intended to end aversive mood states. Accordingly, we expected to find elevated reactions towards palatable food stimuli (Castellanos et al., 2009; Hepworth et al., 2010), especially in a negative mood. For instance, Blechert et al. (2014a) investigated normal-weight participants after several hours (HEE; *M* = 6.79, *SD* = 5.69 and LEE; *M* = 6.81, *SD* = 5.26) of fasting and found negative emotional states to be associated with a heightened craving of high-caloric foods in their pictorial rating task, which was comparable with our experimental procedure aside from not including a nonfood category. However, we observed a preference for sweet over savory food only in a neutral but not in a negative mood, and this preference was bound to a state of self-perceived satiety. Contrary to Blechert et al. (2014a), we did not dichotomize our sample into high and low emotional eaters using a median split on the emotionality scale of the DEBQ (van Strien et al., 1986). According to Bongers and Jansen (2016), increased food intake is not reliably related to the negative mood in individuals with higher scores on emotional eating scales, thereby supporting the proposal that emotional eating might reflect measurements of subjects thinking about their eating rather than their actual eating habits or underlying learned cue-reactivity (cf. Adriaanse et al., 2011). This interpretation might also be reflected by our results, since we assessed momentary perceived food craving as explicit cognitions about visual food stimuli to be strongly influenced by hunger and, to a lesser degree, by mood.

Moreover, our results extend the findings for implicit reactions to food stimuli by Werthmann et al. (2014) to more explicit parts of reactivity. The authors showed that participants in a neutral state of mood compared to those who underwent a negative mood induction revealed an attention bias towards food in a visual probe task. They hypothesized that sad mood might decrease reward sensitivity, an explanation that could also account for our findings. Moreover, our results emphasize that this potential effect might be the cause of differences in food craving, when attention is focused. The more general counterpoint is that the effect of positive emotions might have been underestimated in previous research. However, Evers et al. (2013) showed that a positive mood also increases the ingestion of high-caloric food compared to control conditions. Altogether, emotional eating in terms of addictive behavior theories might reflect a cognitive rather than a physiological process involved in craving. Accordingly, Walsh and Kiviniemi (2014) found evidence of the influence of affective associations on eating behavior. *Via* an implicit priming paradigm, in which pictorial stimuli of fruits were subliminally paired with positive, negative, or neutral affective material (words and pictures), they manipulated participants’ affective associations about fruits. The authors showed that participants in a condition with positive association priming were more likely to select fruits over granola bars after the computerized paradigm compared to participants who underwent negative or neutral priming.

The relationship of hunger and negative affect has been alternatively conceptualized as “feeling hangry,” i.e., hunger seems to shift affective perceptions in a negative context (MacCormack and Lindquist, 2018). The assumed interdependency of hunger and negative mood with regard to eating behavior signifies our consideration that hunger is a highly relevant factor in exploring food craving or similar processes. For instance, the positive association between emotional eating and attachment anxiety was fully mediated by perceived hunger (Alexander and Siegel, 2013).

There are some limitations to our study. First, the sample size of the present study was comparatively small. Therefore, in particular, the three-way interaction has to be carefully interpreted. However, as we realized a variation of hunger and satiety by investigating all subjects twice, our experimental design allowed intraindividual comparisons. Since the order of these compared assessments was not counterbalanced, we cannot exclude consistency effects. Although we chose a long in-between session interval to prevent memory, order, or carry-over effects, we cannot exclude exercise effects with certainty. Second, we included only normal-weight, healthy female subjects. Therefore, our results cannot be generalized to other populations, such as men, non-lean individuals, and those with eating disorders. Hence, future studies should extend our findings to subjects of another gender than female and individuals with obesity and/or eating psychopathologies. To further extend our findings, specifically regarding negative mood induction, it would be of interest to include participants with affective disorders such as depression, in which an increase, as well as a decrease in appetite, is a well-known symptom (e.g., Simmons et al., 2016). Finally, we used a pictorial rating paradigm to assess craving. By doing this, we supposed state food craving to be a result of food cue-reactivity as recently shown in subjects with eating disorders by Meule et al. (2018). To further investigate the specific relationship between state food craving as a physiological reaction at the sight of palatable food (e.g., elevated heart rate, heightened skin conductance; e.g., Nederkoorn et al., 2000), it would be beneficial to combine an experimental setup with the assessment of neurophysiological parameters. Moreover, since our results refer to a group of subjects with low trait food craving (e.g., Brockmeyer et al., 2015), it would be of interest to compare them to participants with high trait food craving levels.

In the present study, we carefully controlled for the impact of self-perceived hunger. This allowed us to identify hunger as a factor of major relevance in food-related research. In summary, we found food craving to be modulated strongly by hunger and to a lesser degree by mood. In line with these findings and bearing all mentioned caveats in mind, it might be beneficial to provide stricter guidelines regarding fasting periods to control participants’ self-perceived hunger in studies implementing food-related stimuli. As mentioned before, studies often have a wide range of fasting periods between subjects and use different instructions to induce hunger. Our procedure of asking participants to follow a period of approximately 6 h of fasting after breakfast with ingestion of a maximum of 2 L of water turned out to be effective. Moreover, given the admittedly smaller effect of mood on food craving, we recommend routinely assessing mood before testing to identify potential confounding effects.

In general, our findings provide the first evidence that testing eating pathology could be specifically interesting and maybe even more valid when patients are sated. The extent to which differences in food saturation might also have an impact on the effect of interventions targeting food craving is unknown. Moreover, the comorbidity of mood disorders and overeating needs a better understanding. Hence, future studies should extend our findings to clinical samples.

## Data Availability Statement

The raw data supporting the conclusions of this article will be made available by the authors, without undue reservation.

## Ethics Statement

Ethical review and approval was not required for the study on human participants in accordance with the local legislation and institutional requirements. The participants provided their written informed consent to participate in this study.

## Author Contributions

JR and AP designed the study and prepared the final version of the manuscript. JR and A-KS conducted literature searches and provided summaries of previous research studies. JR acquired the data. CW provided software support. JR, AP, and CW performed the statistical analyses. All authors contributed to the first draft of the manuscript. AP was supervisor of this project. All authors contributed to the article and approved the submitted version.

### Conflict of Interest

The authors declare that the research was conducted in the absence of any commercial or financial relationships that could be construed as a potential conflict of interest.
